# What has the COVID‐19 pandemic taught us about safety of surgical wait times in urological oncology?

**DOI:** 10.1111/bju.16881

**Published:** 2025-08-05

**Authors:** James P. Blackmur, Chiara Re, Grant D. Stewart

**Affiliations:** ^1^ Department of Urology Western General Hospital Edinburgh UK; ^2^ Institute of Genetics and Cancer, Western General Hospital University of Edinburgh Edinburgh UK; ^3^ Department of Surgery, Cambridge Biomedical Centre University of Cambridge Cambridge UK; ^4^ Department of Urology Addenbrooke's Hospital Cambridge UK; ^5^ Unit of Urology, IRCCS San Raffaele Hospital Vita‐Salute San Raffaele University Milan Italy

**Keywords:** urology, urological neoplasms, urological surgical procedures, COVID‐19, treatment delay

## Abstract

**Objectives:**

To review papers assessing the impact of surgical delay in relation to the COVID‐19 pandemic, and evaluate what has been learnt about the safety of surgical waiting times in urological oncology.

**Patients and Methods:**

Medline and Web of Science were searched for studies published between 1 January 2020 and 1 November 2024. Studies included were those reporting treatment delay effects on surgical or oncological outcomes, or patient experience with reference to COVID‐19. Priority was given to studies deriving their cohort after the start of the pandemic. Studies were also included in which the cohort was derived before the pandemic, but where recommendations were made on COVID‐19 treatment delay. Data were extracted regarding duration of delay and authors’ reported impact of delay on outcome (quantified, and simplified as negative/neutral/positive).

**Results:**

A total of 35 studies met the inclusion criteria. Fourteen studies included data collected after the start of the pandemic and 21 exclusively included cohorts derived prior to the pandemic but made recommendations about COVID‐19‐related treatment delays. Six studies on urothelial cancer reported negative clinical outcomes for delays between 2 weeks and 3 months, while three reported a neutral impact. Four studies on kidney cancer reported negative outcomes with 1–3‐month delay, while two reported a neutral impact. Eleven studies on prostate cancer reported that a 3–12‐month delay had neutral effects, while one reported negative outcomes. One study on penile cancer reported worse survival with delays in treatment. No studies on testicular cancer were identified. Five studies reported negative patient experience with delays, while one reported a positive patient experience.

**Conclusions:**

Few studies have reported the impacts of COVID‐19‐related delayed treatment; this was a missed opportunity. While most prostate cancer treatment can be deferred up to 180 days, diagnostic cystoscopy, transurethral resection of bladder tumour and nephrectomy for cT2+ renal masses should be expedited to occur within 30 days. Treatment of cT1 renal masses, carcinoma invading bladder muscle, upper tract urothelial carcinoma and high‐risk prostate cancer should commence within 90 days.

**Abbreviations**
ADT: androgen deprivation therapy
CWT: Cancer Waiting Time
NCDB: US National Cancer Database
OS: overall survival
TURBT: transurethral resection of bladder tumour

## Introduction

The global COVID‐19 pandemic was a challenge to modern healthcare resources without precedent. From the spring of 2020 until the summer of 2022, countries and healthcare systems around the world struggled not only with treating patients affected by the virus, but also with the challenges of increased demand for hospital beds and of staff absence or redeployment. This significantly affected available surgical resources, particularly in countries which struggled with high infection rates. In the face of this challenge, many countries and hospitals prioritised only the most urgent elective surgery.

In the early months of the pandemic, detailed reviews, based on expert opinion, were published to try to guide triage of surgical procedures [1, 2]. It was recommended that treatment of presumed low‐grade non‐muscle‐invasive bladder cancer could be safely delayed by 3–6 months, while treatment of muscle‐invasive bladder cancer or high‐risk upper tract urothelial carcinoma should occur within 3 months [1, 2]. Intermediate‐ and high‐risk prostate cancer treatment could be deferred by 3–6 months in most cases, and where neoadjuvant androgen deprivation therapy (ADT) is seen as standard pre‐radiotherapy, it was also suggested for consideration pre‐prostatectomy [1, 2]. It was recommended to treat low‐risk prostate cancer by active surveillance. While it was deemed safe to defer the management of cT1‐T2 renal masses (timescale not specified), it was advised to prioritise the management of cT3+ renal masses (timescale not specified) [1, 2]. Prioritisation of inguinal orchidectomy for testicular cancer was also suggested (timescale not specified), along with inguinal lymphadenectomy for penile cancer (within 3 months) [1, 2]. Many reviews did, however, highlight the paucity of data regarding the safety of delayed treatment for many cancer types. Overall, the suggestion was to prioritise interventions according to the prognosis of a patient's cancer and in the context of their comorbidities [3, 4].

Prioritising only the most urgent cases has had a knock‐on effect in the post‐pandemic recovery phase, with large backlogs and delayed presentations. Now almost 3 years on from the lifting of the last restrictions in the UK, there is an opportunity to review the impact of delays in cancer treatment, in a natural history of cancer experiment which has not previously been seen in the modern surgical oncology era. We sought to review papers assessing the impact of surgical delay in relation to the COVID‐19 pandemic, looking at what we have learnt about the safety of surgical waiting times in urological oncology.

## Patients/Materials and Methods

We performed a review focusing on the impact of the COVID‐19 pandemic on wait times in urological oncology. PubMed/Medline and Web of Science were systematically searched for studies published between 1 January 2020 and 1 November 2024. The search strategy used grouped combinations of the following terms: ‘COVID‐19/Pandemic/SARS COV 2/Coronavirus’, ‘Urology/Urological cancer/Bladder cancer/Prostate cancer/Kidney cancer/Testicular cancer’ and ‘Delay/Timing’. We initially included only indexed published articles. Subsequently, additional manuscripts of interest were identified through a manual search of the reference lists of the retrieved articles. The final review represents an overview of what the COVID‐19 pandemic has taught us about the safety of treatment deferral for the most common urological malignancies.

### Inclusion Criteria

We included peer‐reviewed studies that reported on the impact of treatment delay on surgical, pathological or oncological outcome, or patient experience, with reference to the COVID‐19 pandemic. While priority was given to studies that derived their cohort after the start of the pandemic, studies were also included that derived their cohort before the pandemic, but in which recommendations were made on COVID‐19 treatment delay. Papers reporting stage migration during the pandemic, but not reporting the impact of treatment delay on outcome were excluded. We included all original research in English (trials, cohort studies, case–control studies, cross‐sectional studies, qualitative studies, meta‐analyses) that quantified the effects of treatment delay. Editorials, commentaries, authors’ replies, abstracts and case reports were excluded.

After agreement was reached on search terms, one reviewer (J.B.) carried out the search. Title and abstract screening was performed using Rayyan (https://rayyan.ai). Two authors (J.B./C.R.) assessed 10% of titles and abstracts. Once agreement was reached on inclusion/exclusion, full screening of the remaining titles/abstracts was undertaken by one author (J.B.) supported by the Rayyan Artificial Intelligence ‘calculate ratings’ function. Discrepancies were resolved through discussion (J.B./C.R.), with any remaining issues checked by G.D.S. Data were extracted directly into data tables.

### Outcomes

Data on the characteristics of the studies, patients and outcomes were independently extracted by two authors (J.B. and C.R.) according to a predefined form. The following variables were extracted: type of cancer; timing of data collection; number of patients; duration of follow‐up; outcome variables; and reported impact of delay on the specified outcome. The authors’ assessment of the impact of delay was quantified, but also reported as either negative, neutral or positive. Risk of bias was assessed using the ROBINS‐E tool [5].

## Results

**Fig. 1:**
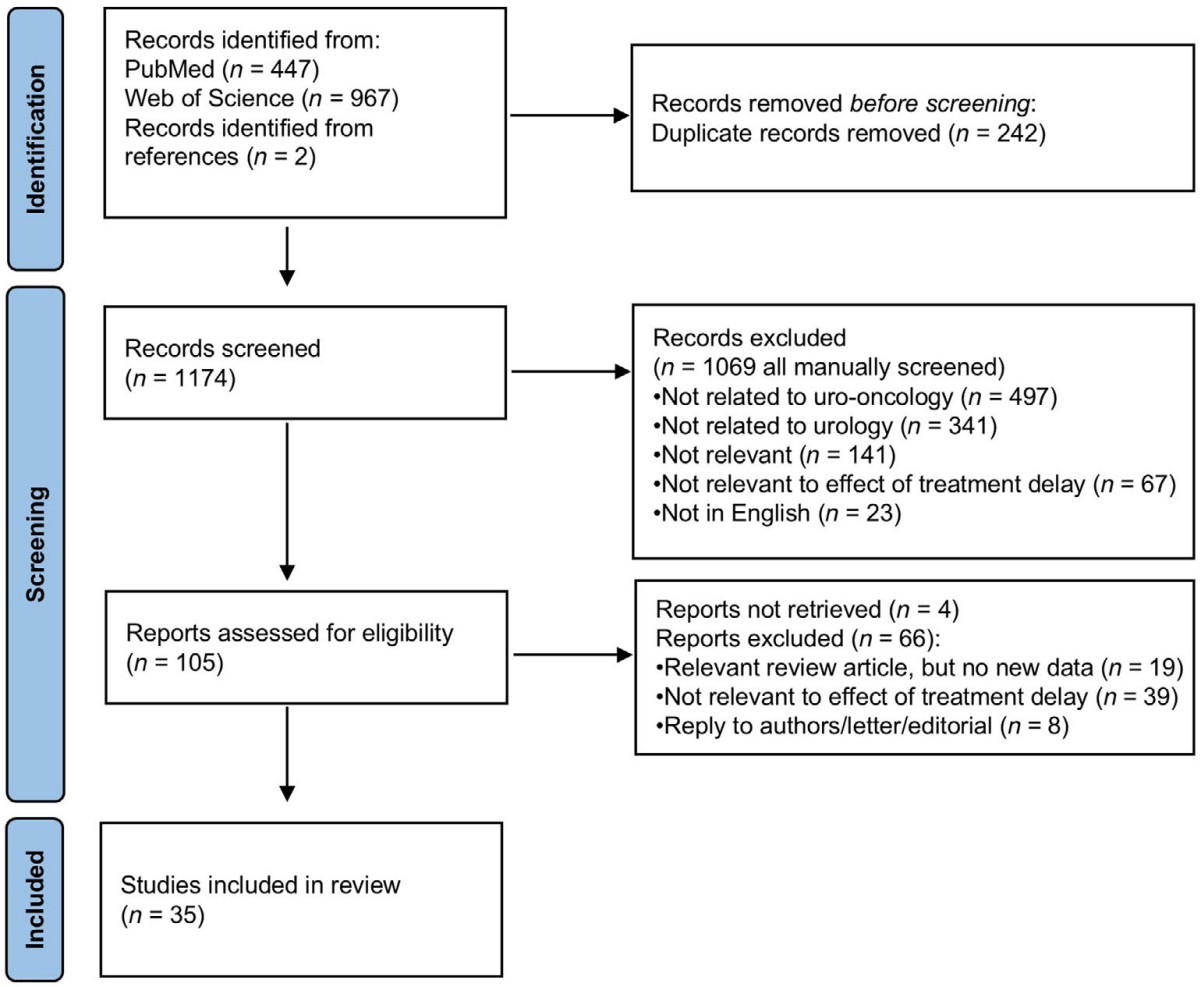
Preferred Reporting Items for Systematic Reviews and Meta‐Analyses (PRISMA) diagram of study inclusions and exclusions.

A total of 1174 papers were identified by the search terms. After exclusions, as per Fig. 1, 35 papers met the inclusion criteria. Eleven studies reported data collected after the start of the COVID‐19 pandemic and three reported data collected both before and after the start of the pandemic. These 14 studies are summarised in Table 1. Twenty studies exclusively reported data from cohorts derived prior to the COVID‐19 pandemic, and one study did not state the period over which data were collected (summarised in Table S1). Risk of bias is shown in Figure S1.

**Table 1:** Summary of studies reporting the impacts of treatment delay, where some or all of the study's cohort was derived after the onset of the COVID‐19 pandemic.

Author	Cancer type	Cohort	Years cohort derived from	Patients, *N*	Duration of follow‐up	Delay	Actual outcome
Li et al. 2022 [19]	Bladder MIBC	Two centres, China	2008–2020	165	Median 48 months	≤30 days, 31–90 days and >90 days RC	Delay >90 days associated with worse OS and CSS: 5‐year OS long, intermediate and short waiting time groups 35.7%, 61.3% and 64.1%. 5‐year CSS 38.9%, 61.5%, and 65.0%
Culpan et al. 2021 [20]	Bladder NMIBC	Multicentre, Turkey	2020	407	Not stated	2‐5‐month delay to follow‐up cystoscopy	Delay increased risk of recurrence (OR 2.4) and progression (OR 6.7) in multivariable models
Ok et al. 2022 [21]	Bladder NMIBC	Single‐centre, Turkey	2017–2022	93	Not stated	Delay from symptoms to diagnosis (pre‐COVID median 5 week vs post‐COVID 7 week), and delay to TURBT (2 week vs 3 week)	Increased risk recurrence post‐COVID: 13.3% vs 33.3%. Time from symptoms to diagnosis >7.5 weeks associated with increased risk of recurrence
Alexander et al. 2024 [22]	Bladder NMIBC	Multicentre, international	2020	229	1 year	Investigator reported delay to TURBT or change in usual treatment (e.g. re‐resection). Duration not stated	‘Delay’ resulted in higher recurrence (29.3%) and progression rates (9.7%)
Staehler et al. 2021 [16]	Kidney	KCCure survey	2020	539	NA	Not stated	65% patients with localised RCC unwilling to delay follow‐up scans; 51% with mRCC preferred not to defer SACT
Campi et al. 2021 [17]	Multiple: prostate, urothelial and kidney	Single‐centre, Italy	2020	171	NA	Not stated	87% patients preferred delay to treatment in context of COVID‐19
Glick et al. 2022 [14]	Multiple: prostate, bladder and kidney	Single‐centre, USA	2020	18	NA	Not stated	Patient concern about risk of progression, and concern with cancer surgery labelled ‘elective’, but concerns could be mitigated by good communication
Tiwari et al. 2024 [23]	Multiple	Multicentre, UK	2006–2022	4124	16 years	NA	Successful claims increased 2.9‐fold. Largest increase 2020–2022. Failure or delay to treatment 21% of claims; failure or delay to diagnosis 15% of claims
Faris et al. 2022 [15]	Multiple: prostate, MIBC, or advanced renal cancer	Midwestern Cancer Center and the Bladder Cancer Advocacy Network, USA	2020	45	NA	Median 6 months since diagnosis	Patient concern about risk of progression. Relief at avoiding COVID exposure
Ian Janes et al. 2023 [24]	Penile	Single‐centre, Canada	2020–2022	11	Not stated	Mean 75 days symptom onset to surgery	60% patients inguinal disease, indicative of late presentation
Kizilkan et al. 2021 [25]	Prostate	Single‐centre, Turkey	2020	24	NA	Mean 79 days since diagnosis	Anxiety and mild depression related to treatment delay
Sokas et al. 2021 [13]	Prostate	Single centre, USA	2020	13	NA	Not stated	Patient concern about treatment delays, and of potential exposure to COVID infection
Bennett et al. 2023 [26]	Prostate	Single centre, UK	2019–2020	124	NA	Mean 16 weeks on waiting list for prostatectomy COVID cohort vs 5 weeks pre‐COVID	Patients with delayed surgery given neoadjuvant ADT trend to reduction in PSMs, N+ disease and pathological downstaging (50% patients with pT2 disease)
Nathan et al. 2024 [27]	Prostate	Multicentre, International	2020	467	1 year	Investigator reported delay to prostatectomy (duration not stated)	Delay not associated with PSM, BCR or upgrading. Delay associated with risk upstaging (35% vs 49%)

Colour coding indicates impact of treatment delay within the study's timescale: negative (red), neutral (yellow) and positive (green). ADT, androgen deprivation therapy; BCR, biochemical recurrence; CSS, cancer‐specific survival; MIBC, muscle invasive bladder cancer; mRCC, metastatic renal cell carcinoma; N+, node‐positive disease; NMIBC, non‐muscle invasive bladder cancer; OR, odds ratio; OS, overall survival; PSM, positive surgical margin; RC, radical cystectomy; SACT, systemic anti‐cancer therapy; TURBT, transurethral resection of bladder tumour.

Overall, 10 of 14 studies on prostate cancer reported that a treatment delay of between 3 and 12 months was not associated with a range of outcomes including pathological upstaging, biochemical recurrence and overall survival. One prospective study reported that investigator‐perceived COVID‐19‐associated treatment delay (duration not specified) was associated with risk of upstaging, but not biochemical recurrence at 12 months. While one meta‐analysis reported worse outcomes with delays to prostatectomy over 5 months, 4 months or 30 days for low‐risk, intermediate‐risk, and high‐risk disease, respectively, this was not demonstrated in other large registry studies. The risks of delayed treatment also appeared to be mitigated somewhat by neoadjuvant ADT. Two studies reported that patient anxiety was associated with treatment delay.

For patients with urothelial cancer, studies including post‐COVID‐19 cohorts assessed impacts of delay to cystoscopy and transurethral resection of bladder tumour (TURBT), while studies from pre‐COVID‐19 cohorts reviewed the impact of delay to radical cystectomy, chemoradiotherapy or radical nephroureterectomy. Time from symptom onset to diagnostic cystoscopy over 7.5 weeks, perceived delay to TURBT and delays over 2 months for follow‐up cystoscopy resulted in increased risk of recurrence and progression. Delay to cystectomy over 3 months was associated with worse cancer‐specific survival and overall survival (OS), although the impact could potentially be mitigated by neoadjuvant chemotherapy. For patients with upper tract urothelial carcinoma, delay to performing radical nephroureterectomy of over 3 months was associated with worse OS.

There have been no studies published on the direct impact of COVID‐19 delay on pathological or oncological outcomes for patients with kidney cancer. One survey reported that patients were unwilling to delay follow‐up imaging during the pandemic, but all other studies derived their cohort exclusively from the pre‐COVID‐19 period. In those studies, the impact of delay on outcome was mixed; delay to treatment of cT1 tumours of up to 3 months showed no impact on OS, while for patients with cT2+ tumours, delay over 30 days was associated with worse OS.

One small, single‐centre study in patients with penile cancer reported that, in 2020–2021, 60% of patients had inguinal disease which was seen as indicative of late presentation. The mean delay from symptom onset to diagnosis in 2020 was 62 days, compared to 18 days in 2021, although further analysis of oncological outcomes was not provided. Similarly, no study has reported outcomes for delayed treatment of testicular cancer.

Two studies of patient experience across prostate, bladder and kidney cancers reported concern about the risk of progression associated with treatment delay, while one similar study reported that patients preferred delay to treatment in the context of possible COVID‐19 infection. One study assessed the cost of litigation over several years, with the largest increase in successful claims seen in the period 2020–2022, and with delayed diagnosis and delayed treatment accounting for 36% of claims.

## Discussion

This review highlights that there have been few studies reporting the impact of COVID‐19‐related delay on patients with urological cancers. As most of the pre‐COVID‐19 studies are registry‐based, with many derived from the US National Cancer Database (NCDB), the reasons for treatment delay may be heterogeneous: some patients will have been deliberately placed on active surveillance strategies, while others may have had significant comorbidities or concurrent pathology precluding treatment. Of the 14 studies that derived some or all of their cohort after the onset of the pandemic, six reported on the impact of delay on patient anxiety and one reviewed litigation costs. Only seven of 14 studies reported the pathological or oncological impact of treatment delay. The definition of what constitutes ‘delay’ was also highly variable, with some studies reporting impacts on survival associated with a 20‐day delay, while others considered delay up to 1 year.

In the early stages of the pandemic, many organisations rapidly produced guidelines based on expert opinion regarding which cancer surgery could be delayed [2, 6]. This review highlights that there has been little published on the extent to which these delays were required in different healthcare settings around the world, and on the impacts of those delays. Did providing ADT for patients with prostate cancer have a beneficial effect, particularly when there was a delay to surgery? Did prioritisation of cT3+ renal cancer result in better outcomes for those patients, with no impact on those with cT1 disease? These questions remain open. There are numerous studies reporting stage migration during the pandemic [7, 8, 9, 10, 11, 12], largely because of cessation of screening programmes, and patient avoidance of hospital resulting in detection of fewer incidental cancers. As the natural history of many (particularly early‐stage) cancers is prolonged, there is an opportunity to continue to review the impact of delays in presentation and treatment. A few multicentre studies highlighted in Table 1 have published short‐term outcomes for patients diagnosed with prostate and urothelial cancers during the pandemic, and continuing to follow these patients will provide valuable insights into the impacts of delayed treatment. There is a clear absence of data regarding the impact of COVID‐19 delays on outcomes for patients with kidney cancer (particularly cT1 renal masses), and for those with testicular or penile cancers. National datasets, such as the National Kidney Cancer (https://www.natcan.org.uk/audits/kidney/) or Prostate Cancer audits (https://www.natcan.org.uk/audits/national‐prostate‐cancer‐audit/) in the UK and the NCDB, may provide valuable insights into the natural history of cancers in the modern era. Similarly, there is much to learn from comparison of outcomes among countries which prioritised cases in different ways. The value of those datasets will however be determined by the availability of individual‐level data, particularly on time from referral or investigation to definitive treatment.

While it may be deemed ‘safe’ to delay treatment, multiple studies highlight that patients may have significant anxiety [13, 14, 15]. This anxiety could be alleviated by good patient–physician communication and the presence of clear strategies for management of delays [13, 15, 16]. Patient perception of the risk of delay or the risk of COVID‐19 infection was predictably influenced by patient age, fitness and the nature of the underlying disease [17].

In particular, the review highlights that not all cancers are equal, and not all patients with cancer will be affected negatively by delays in treatment. The pandemic provided an opportunity to rationalise and re‐prioritise activities, but it is doubtful whether clinicians have been given enough bandwidth to consider what might be done differently. In the UK, the Cancer Waiting Time (CWT) pathway specifies that patients should wait no more than 14 days between referral and first review by a specialist, 28 days between referral and diagnosis and 62 days between referral and starting treatment [18]. Such targets are blunt instruments – for example, prostate and pancreatic cancers are considered identically – and perhaps a more nuanced tool for cancer‐tracking would be more useful. However, given that adherence to CWT targets is included in key performance indicators, political willingness and interest would be required to move to a more nuanced approach. If there are limits to available surgical resources, does it make sense for patients with the highest‐risk cancers (e.g. T3b + renal masses, muscle‐invasive bladder cancer, penile cancer) to be on the same pathway as those with a small renal mass or low‐risk prostate cancer? In this space, we propose recommended cancer waiting times as per Table 2, corrected for the available data regarding the impact of treatment delay.

**Table 2:** Proposed cancer waiting time targets corrected for differences in impact of delay on outcome per cancer type.

Cancer type	Treatment	Recommended treatment time
Bladder	Diagnostic cystoscopy	<30 days from symptom onset
	TURBT	<30 days from diagnosis
	Radical cystectomy or radiotherapy (with consideration of neoadjuvant chemotherapy)	<90 days from diagnosis
Upper tract urothelial carcinoma	Radical nephroureterectomy	<90 days from diagnosis
Kidney	Nephrectomy cT2+ renal mass	<30 days from diagnosis
	Treatment cT1 renal mass	<90 days from diagnosis (with consideration of active surveillance)
Prostate	Radical prostatectomy or radiotherapy (with ADT) for high‐risk prostate cancer	<90 days from diagnosis
	Radical prostatectomy or radiotherapy (with ADT) for intermediate‐ or low‐risk prostate cancer	<180 days from diagnosis (with consideration of active surveillance)
Penile	Unable to make recommendation: early treatment recommended	
Testicular	Unable to make recommendation: early treatment recommended	

ADT, androgen deprivation therapy; TURBT, transurethral resection bladder tumour.

The COVID‐19 pandemic also saw a significant impact on benign urological conditions, and while those may not be subject to the same waiting time targets, the impact of delay may also be harmful. The start of the pandemic saw guidelines recommending delay of surgery for BOO, urethral strictures, routine stent changes and almost all female, reconstructive and andrological surgeries [6]. Along with reviewing the impacts of treatment delay in urological oncology, there is also an opportunity to review how benign urological procedures should be prioritised within the structure of Table 2 above. Is it right that patients with low‐risk cancers such as small renal masses or localised prostate cancer be prioritised over patients with limiting benign conditions, such as patients with long‐term catheters awaiting bladder outflow surgery?

In conclusion, although numerous guidelines and reviews, based on expert opinion, were published at the start of the COVID‐19 pandemic suggesting which treatments could be delayed, there have been few studies published reporting the impact of delayed treatment, and of delayed presentation; this was a missed opportunity. However, those patient cohorts that were reported on provide an interesting insight into the natural history of many cancer types, and there is an opportunity to review these data with an eye towards re‐prioritisation and redesign of resources.

## Disclosure of Interests

J.P.B. and C.R. have no conflicts of interest to declare. G.D.S. has received educational grants from AstraZeneca, consultancy fees from Evinova, and travel expenses from MSD, and is Clinical lead (urology) National Kidney Cancer Audit and Topic Advisor for the NICE kidney cancer guideline.

## Supporting information


**Table S1.** Summary of studies reviewing impact of treatment delay, and where the cohort was derived exclusively prior to the onset of the COVID Pandemic.
**Fig. S1.** Risk of bias assessment of included studies.
