# Supplementary material for: What has the COVID‐19 pandemic taught us about safety of surgical wait times in urological oncology?
Source: BJU Int. 2025 Aug 5;136(5):781–7. doi: 10.1111/bju.16881 (PMC12522522; doi:10.1111/bju.16881)
Supplement: Supplementary file 1 — Table S1. Summary of studies reviewing impact of treatment delay, and where the cohort was derived exclusively prior to the onset of the COVID Pandemic. Fig. S1. Risk of bias assessment of included studies. [file BJU-136-781-s001.docx]

Supp Table 1: Summary of studies reviewing impact of treatment delay, and where the cohort was derived exclusively prior to the onset of the COVID Pandemic. Colour coding indicates impact of treatment delay within the study’s timescale: negative (red), neutral (yellow) and positive (green).

d day, w Week, m Month, yr Year, MIBC muscle invasive bladder cancer, OS Overall survival, DFS disease-free survival, CSS Cancer-specific survival, RNU radical nephroureterectomy, TURBT transurethral resection of bladder tumour, RC Radical cystectomy, BCR biochemical recurrence, PSM positive surgical margin, N+ node positive disease, UTUC upper tract urothelial carcinoma, NCDB US National Cancer Database, NAC neoadjuvant chemotherapy, PCa prostate cancer, ADT androgen deprivation therapy, CRPC castrate-resistant prostate cancer.

| Author | Cancer Type | Cohort | Years cohort derived from | N Patients | Duration follow up | Delay | Actual outcome |
| --- | --- | --- | --- | --- | --- | --- | --- |
| Fischer-Valuck BW et al 2021(1) | Bladder MIBC | NCDB US registry | 2004-2015 | 1584 | Median OS in outcome | ≤90d vs >90d TURBT to chemoradiotherapy | No difference OS between cohorts (median 29m vs 27m) |
| Kotha NV et al 2022(2) | Bladder MIBC | Veterans' Affairs USA | 2000-2018 | 305 | Median 75m | ≤90d vs >90d Chemoradiotherapy | No impact local/regional or distant failure |
| Leow J et al 2022(3) | Bladder & UTUC | Meta-analysis | 1982-2016 | 12201 bladder 4629 UTUC | Various | 60-90d delay RC/RNU | Delay TURBT to RC worse OS (HR 1.25); Delay NAC to RC no impact OS; Delay diagnosis to RNU worse OS (HR 1.55) and CSS (HR 2.55) |
| Lee HY et al 2021(4) | UTUC | Multicentre Hong Kong and Taiwan | 2000-2019 | 665 | Median 40m | ≤3m vs >3m delay RNU | RNU >3m worse OS (HR 1.55) |
| Zhao F et al 2021(5) | UTUC | NCDB US registry | 2001-2010 | 3581 | Median 40.4m | ≤30d, 31-90d, >90d delays RNU | No effect waiting time up to 120d on OS |
| Tan WS et al 2021(6) | Kidney | NCDB US registry | 2004-2015 | 92586 T1; 20564 T2+ | Median 39m | ≤30d, 31-90 and >90d delay surgery | T1a and b, no impact on OS by delay ≤90d, but worse OS >90d (T1a ≤30d 16.2% *vs.* 31-90d 15.9% vs >90d 19.5%; T1b ≤30d 22.9% *vs.* 31-90d 23.8% vs >90d 30.3%).  T2 worse OS if delay >30d (≤30d 31.6% *vs.* 31-90d 34.2% vs >90d 38.1%) |
| Ou W et al 2022(7) | Kidney | Multicentre China | 2004-2015 | 428 T1a; 334 T1b | Median 44m | 5wk+ delay surgery | T1a RCC no impact DFS or OS. T1b RCC delay shorter DFS (HR 2.9) and OS (HR 2.5) |
| Celik S et al 2023(8) | Kidney | Multicentre Turkey | 2007-2019 | 3258 | Not stated | Mean 49d (SD 97) delay surgery | Optimal time for surgery <30d T1 and <20d T2-T4 tumours |
| Srivastava A et al 2021(9) | Kidney T1b-T2b | NCDB US registry | 2004-2014 | 29746 | NA | ≤90d vs >90d delay surgery | Delay >90d did not increase risk pT3a upstaging |
| Chan VW et al 2021(10) | Kidney | Meta-analysis | 1988-2018 | 29274 | Various | Various delays treatment | Delay associated with worse CSS for cT1a (HR 1.67), but no impact OS. Insufficient data for cT1b+ |
| Ginsburg KB et al 2021(11) | Kidney T2 | NCDB US registry | 2004-2015 | 11848 | 42m | 1-2m vs 3-4m or 5-6m delay surgery | Worse OS with delay (3-4m HR 1.12, 5-6m HR 1.51). In healthy individuals, worse OS delay 5-6m (HR 1.68) |
| Cone EB et al 2020(12) | Prostate | NCDB US registry | 2004-2015 | 853030 | 5 and 10yr | Time to prostatectomy 61-120d vs 181-365d | Prostate cancer least affected by treatment delay: 5yr predicted mortality for high risk PCa 61-120d 12.8% vs 181-365d 14.1% |
| Dee EC et al 2020(13) | Prostate | NCDB US registry | 2004-2014 | 63858 | 10yr OS | Time RT relative to ADT: 0-60days prior to ADT, 0-60d after, 61-120d after, 121-180d after | No effect on OS |
| Ginsburg KB et al 2020(14) | Prostate Intermediate-/High-risk | NCDB US registry | 2010-2016 | 128062 | NA | 3m cohorts delay prostatectomy up to 12m | No effect delay on adverse pathology, upgrading, N+ disease or need for secondary treatments |
| Chan VW et al 2021(15) | Prostate | Meta-analysis | 1967-2018 | 127597 | Various | Various delays to prostatectomy | Delays over 5m for low-risk, 4m intermediate-risk, and 30d high-risk PCa associated with worse pathological and oncological outcomes |
| Diamand R et al 2021(16) | Prostate | Multicentre Europe | 2012-2019 | 926 | Median 26m | Median 3m delay prostatectomy | No effect delay on upstaging (pT3/T4 or pN+) or BCR |
| Lee MC et al 2022(17) | Prostate | Veterans' Affairs USA | 1988-2018 | 3962 | Median 84 and 87m (intermediate and high risk cohorts) | Biopsy to prostatectomy <3m, 3-6m, >6m | No increased risk of CRPC, distant recurrence or OS with delays up to 1 year |
| Mohamad O et al 2024(18) | Prostate | Meta-analysis of RCTs | 1988-2016 | Not stated | Variable | Various delays to prostatectomy or radiotherapy | Delay 3-8 months RP or RT safe (impact on BCR, CSS, OS beneficial for RT) if neoadjuvant ADT provided |
| Sahin B et al 2024(19) | Prostate | Single centre Turkey | Not stated | 2454 | NA | ≤90d vs >90d delay prostatectomy | No effect delay on adverse pathology or BCR |
| Xia L et al 2020(20) | Prostate High-risk | NCDB US registry | 2006-2016 | 32184 | Median 41.7m | 30d cohorts up to 180d delay prostatectomy | No effect on pT3/4 disease, pN+ disease, PSM, or OS |
| Laukhtina E et al 2021(21) | Prostate Intermediate-/High-risk | Meta-analysis | 2011-2020 | 163576 | Various | 3m delay prostatectomy | No effect delay on upstaging or survival |

Supplementary figure 1 Risk of bias assessment of included studies


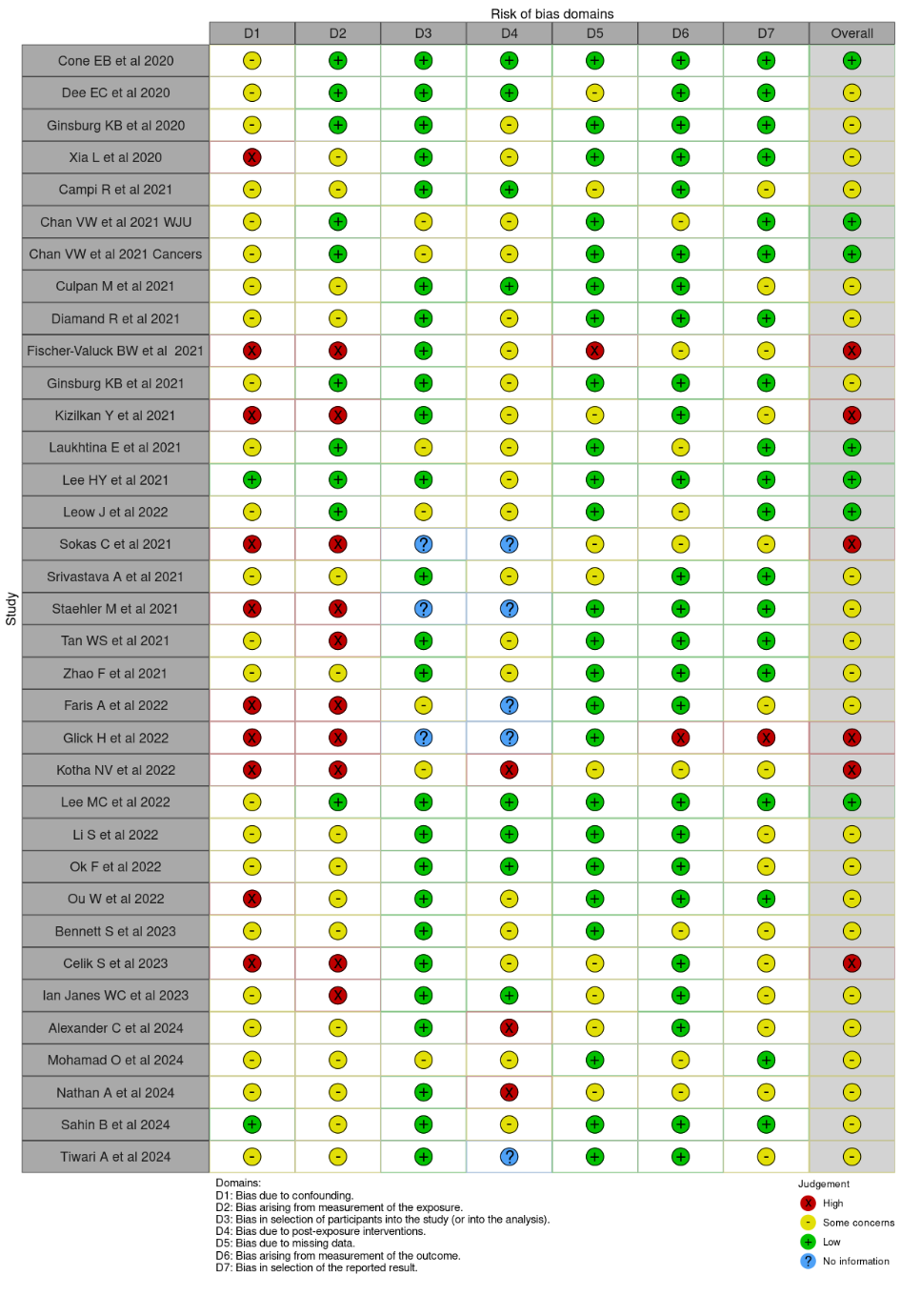


References

1. Fischer-Valuck BW, Michalski JM, Harton JG, Birtle A, Christodouleas JP, Efstathiou JA, et al. Management of Muscle-Invasive Bladder Cancer During a Pandemic: Impact of Treatment Delay on Survival Outcomes for Patients Treated With Definitive Concurrent Chemoradiotherapy. Clin Genitourin Cancer. 2021 Feb;19(1):41-46.e1.

2. Kotha N V, Kumar A, Nelson TJ, Qiao EM, Qian AS, Voora RS, et al. Outcomes by time to definitive chemoradiation treatment for patients with muscle-invasive bladder cancer. Urol Oncol. 2022 Jun;40(6):274.e1-274.e6.

3. Leow JJ, Tan WS, Tan WP, Tan TW, Chan VWS, Tikkinen KAO, et al. A systematic review and meta-analysis on delaying surgery for urothelial carcinoma of bladder and upper tract urothelial carcinoma: Implications for the COVID19 pandemic and beyond. Front Surg. 2022;9:879774.

4. Lee HY, Chan EOT, Li CC, Leung D, Li WM, Yeh HC, et al. How to manage patients with suspected upper tract urothelial carcinoma in the pandemic of COVID-19? Urol Oncol. 2021 Oct;39(10):733.e11-733.e16.

5. Zhao F, Qi N, Zhang C, Xue N, Li S, Zhou R, et al. Impact of Surgical Wait Time on Survival in Patients With Upper Urinary Tract Urothelial Carcinoma With Hydronephrosis. Front Oncol. 2021;11:698594.

6. Tan WS, Marchese M, Paciotti M, Nguyen DD, Cone EB, Mossanen M, et al. Delay in surgery for cT1b-2 kidney cancer beyond 90 days is associated with poorer survival: implications for prioritization during the COVID-19 pandemic. Minerva urology and nephrology. 2021 Jun;73(3):404–6.

7. Ou W, Wang C, Un H, Guo S, Xiao H, Huang B, et al. Impact of Time-To-Surgery on the Prognosis of Patients with T1 Renal Cell Carcinoma: Implications for the COVID-19 Pandemic. J Clin Med. 2022 Dec 19;11(24).

8. Çelik S, Tinay İ, Sözen S, Özen H, Akdoğan B, Aslan G, et al. What is the Optimal Time Period for Postponing Nephrectomy in Patients with Renal Cell Carcinoma of Various Stages? The Bulletin of Urooncology. 2023 Dec 4;22(4):150–5.

9. Srivastava A, Patel H V, Kim S, Shinder B, Sterling J, Tabakin AL, et al. Delaying surgery for clinical T1b-T2bN0M0 renal cell carcinoma: Oncologic implications in the COVID-19 era and beyond. Urol Oncol. 2021 May;39(5):247–57.

10. Chan VWS, Tan WS, Leow JJ, Tan WP, Ong WLK, Chiu PKF, et al. Delayed surgery for localised and metastatic renal cell carcinoma: a systematic review and meta-analysis for the COVID-19 pandemic. World J Urol. 2021 Dec;39(12):4295–303.

11. Ginsburg KB, Curtis GL, Patel DN, Chen WM, Strother MC, Kutikov A, et al. Association of Surgical Delay and Overall Survival in Patients With T2 Renal Masses: Implications for Critical Clinical Decision-making During the COVID-19 Pandemic. Urology. 2021 Jan;147:50–6.

12. Cone EB, Marchese M, Paciotti M, Nguyen DD, Nabi J, Cole AP, et al. Assessment of Time-to-Treatment Initiation and Survival in a Cohort of Patients With Common Cancers. JAMA Netw Open. 2020 Dec 1;3(12):e2030072.

13. Dee EC, Mahal BA, Arega MA, D’Amico A V, Mouw KW, Nguyen PL, et al. Relative Timing of Radiotherapy and Androgen Deprivation for Prostate Cancer and Implications for Treatment During the COVID-19 Pandemic. JAMA Oncol. 2020 Oct 1;6(10):1630–2.

14. Ginsburg KB, Curtis GL, Timar RE, George AK, Cher ML. Delayed Radical Prostatectomy is Not Associated with Adverse Oncologic Outcomes: Implications for Men Experiencing Surgical Delay Due to the COVID-19 Pandemic. J Urol. 2020 Oct;204(4):720–5.

15. Chan VWS, Tan WS, Asif A, Ng A, Gbolahan O, Dinneen E, et al. Effects of Delayed Radical Prostatectomy and Active Surveillance on Localised Prostate Cancer—A Systematic Review and Meta-Analysis. Cancers (Basel). 2021 Jun 30;13(13):3274.

16. Diamand R, Ploussard G, Roumiguié M, Oderda M, Benamran D, Fiard G, et al. Timing and delay of radical prostatectomy do not lead to adverse oncologic outcomes: results from a large European cohort at the times of COVID-19 pandemic. World J Urol. 2021 Jun;39(6):1789–96.

17. Lee MC, Erickson TR, Stock S, Howard LE, De Hoedt AM, Amling CL, et al. Association between Delay to Radical Prostatectomy and Clinically Meaningful Outcomes among Patients with Intermediate and High-Risk Localized Prostate Cancer. J Urol. 2022 Mar;207(3):592–600.

18. Mohamad O, Li YR, Feng F, Hong JC, Wong A, El Kouzi Z, et al. Delayed definitive management of localized prostate cancer: what do we know? Prostate Cancer Prostatic Dis. 2024 Aug 11;

19. Sahin B, Bozkurt O, Sözen S, Ozen H, Akdogan B, Aslan G, et al. LESSONS FOR COVID 19 ERA: IMPACT OF DELAYS IN SURGERY ON BIOCHEMICAL RECURRENCE-FREE SURVIVAL AND ADVERSE ONCOLOGICAL OUTCOMES IN PROSTATE CANCER PATIENTS. 2021.

20. Xia L, Talwar R, Chelluri RR, Guzzo TJ, Lee DJ. Surgical Delay and Pathological Outcomes for Clinically Localized High-Risk Prostate Cancer. JAMA Netw Open. 2020 Dec 1;3(12):e2028320.

21. Laukhtina E, Sari Motlagh R, Mori K, Quhal F, Schuettfort VM, Mostafaei H, et al. Oncologic impact of delaying radical prostatectomy in men with intermediate- and high-risk prostate cancer: a systematic review. World J Urol. 2021 Nov;39(11):4085–99.
